# Biasing the Hierarchy Motifs of Nanotoroids: from 1D Nanotubes to 2D Porous Networks

**DOI:** 10.1002/anie.202114290

**Published:** 2021-12-15

**Authors:** Jorge S. Valera, Hironari Arima, Cristina Naranjo, Takuho Saito, Natsuki Suda, Rafael Gómez, Shiki Yagai, Luis Sánchez

**Affiliations:** ^1^ Dpto. Química Orgánica Facultad de Ciencias Químicas Universidad Complutense de Madrid Ciudad Universitaria, s/n 28040 Madrid Spain; ^2^ Division of Advanced Science and Engineering Graduate School of Science and Engineering Chiba University 1–33, Yayoi-cho, Inage-ku Chiba 263-8522 Japan; ^3^ Department of Applied Chemistry and Biotechnology Graduate School of Engineering Chiba University 1–33, Yayoi-cho, Inage-ku Chiba 263-8522 Japan; ^4^ Institute for Global Prominent Research (IGPR) Chiba University 1–33, Yayoi-cho, Inage-ku Chiba 263-8522 Japan

**Keywords:** azobenzene, hierarchical organization, nanotoroids, photoresponsive systems, supramolecular polymers

## Abstract

Hierarchical organization of self‐assembled structures into superstructures is omnipresent in Nature but has been rarely achieved in synthetic molecular assembly due to the absence of clear structural rules. We herein report on the self‐assembly of scissor‐shaped azobenzene dyads which form discrete nanotoroids that further organize into 2D porous networks. The steric demand of the peripheral aliphatic units diminishes the trend of the azobenzene dyad to constitute stackable nanotoroids in solution, thus affording isolated (unstackable) nanotoroids upon cooling. Upon drying, these nanotoroids organize at graphite surface to form well‐defined 2D porous networks. The photoirradiation with UV and visible light enabled reversible dissociation and reconstruction of nanotoroids through the efficient trans↔cis isomerization of azobenzene moieties in solution.

## Graphical abstract

The steric demand exerted by the peripheral side chains of the reported azobenzene dyads impedes their hierarchical self‐assembly from nanotoroids into nanotubes. In contrast, they organize into 2D porous networks on substrate upon solution processing. Photoisomerization of azobenzene moieties allows efficient dissociation/reorganization of nanotoroids in solution, demonstrating their potential as photoresponsive 2D porous nanosheets.

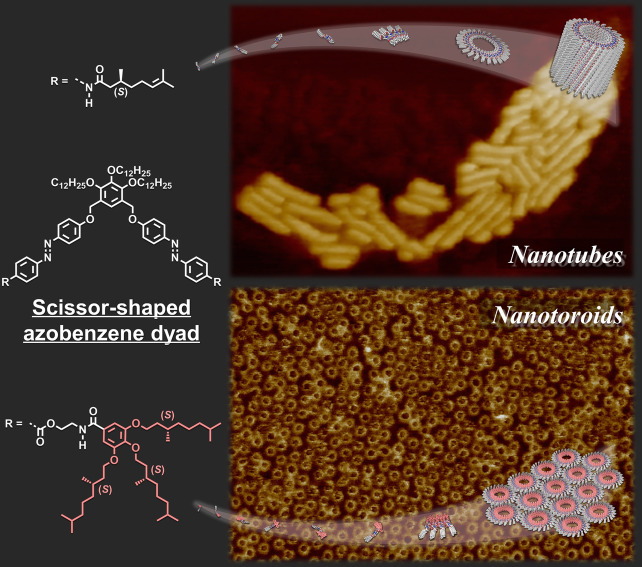

## Introduction

Complex self‐assembly processes, in which relatively simple molecular scaffolds are able to generate intricate and functional supramolecular structures, are ubiquitous in Nature and constitute a source of inspiration for supramolecular chemists.^[1]^ Collagen, a coiled‐coil superhelical structure of proteins responsible for providing strength and resistance to cartilage and bone, is an excellent example of self‐assembled structure showing different levels of hierarchy.^[2]^ The formation of collagen fibers, as many other natural and man‐made hierarchical self‐assembled systems, involves the interaction of one‐dimensional (1D) fibers into thicker filaments that, in turn, exhibit helical character.^[3]^ A number of these 1D supramolecular structures have found applicability as new materials with enhanced mechanical, biomedical or optoelectronic properties.^[4]^ However, the hierarchical organization of self‐assembled structures to give rise to two‐dimensional (2D) molecular networks on surfaces is also a stimulating research field due to the void spaces generated, that enables the complexation of complementary guests, among other applications.^[5]^ A number of examples of 2D porous networks relies on the physisorption of covalent macrocycles that requires previous, time‐consuming synthetic protocols to be achieved.^[6]^ The supramolecular approach is an attractive alternative to attain 2D porous networks. The physisorption of suitable scaffolds, able to interact by multiple non‐covalent forces to result in toroidal nanostructures with different size and shape,^[7]^ represents an emerging field not yet well‐explored since the weak and dynamic character of the non‐covalent forces joining together the molecular building blocks limit the control on the targeted 2D network.^[8]^ Noteworthy, this toroidal topology is present in complex, functional natural structures like the photosynthetic apparatus.^[9]^

On the other hand, several applications shown by man‐made supramolecular polymers stem from the responsiveness of some of these materials towards different stimulus, being light one of the most explored.^[10]^ Recently, the scope of these macromolecules has been widen with the advent of seeded and living supramolecular polymerization, which have enabled to accurately control the size, growth and monodispersity of 1D and 2D supramolecular polymers.^[11]^ This milestone is a consequence, among other factors, of the understanding of the molecular design required to rule different aggregation pathways.^[12]^ However, structural rules leading to the hierarchical organization of self‐assembled structures towards well‐defined superstructures are less intelligible.^[13]^ To attain this goal, more specific rules that establish the relation between molecular design, supramolecular structure and organization of these self‐assembled entities are required.[^14^, ^15^, ^16^]

**Figure 1:**
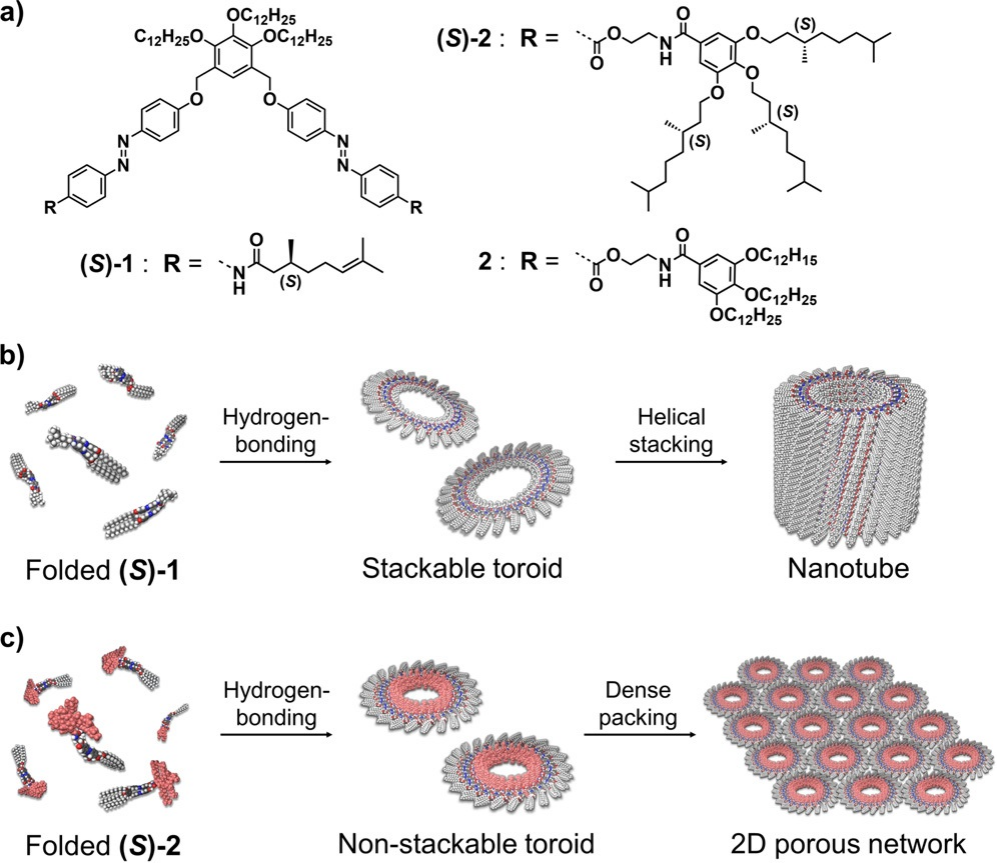
a) Molecular structures of scissor‐shaped azobenzene dyads **(*S*)‐1**, **(*S*)‐2** and **2**. b),c) Representations of the self‐assembly of b) **(*S*)‐1** and c) **(*S*)‐2** and **2**.

In this context, we have previously reported the self‐assembling features of scissor‐shaped azobenzene dyad **(*S*)‐1**,^[15]^ endowed with hydrophobic side chains, which is capable of experiencing self‐assembly at different levels of hierarchy (Figure 1 a). In methylcyclohexane (MCH) at 20 °C, this scissor‐shaped dyad self‐assembles into homogenous toroidal nanostructures (nanotoroids). These nanotoroids further stack into nanotubes upon cooling the solution to 0 °C. Such a discrete nature is in contrast to other circular supramolecular polymers.^[14]^ The distinctive self‐assembly process of **(*S*)‐1** is ascribable to the intramolecular folding through the π‐stacking of two azobenzene moieties and the hydrogen‐bonding between the amide groups to produce spontaneous curvature thus yielding well‐defined, homogenous toroidal structures (Figure 1 b). Notably, the nanotube of **(*S*)‐1** can be reversibly dissociated and reconstructed by making use of the photoisomerization processes experienced by azobenzene moieties. The uniqueness of the self‐assembled nanotoroids and the precise hierarchical process of organization motivated us to investigate further ways of modifying and organizing discrete nanotoroids into new and different arrangement patterns.

Thus, in an attempt to unravel clear rules in the structure‐function correlation of these discrete moieties, we envisioned that replacing the originally reported peripheral side chains with more sterically demanding peripheral side wedges could strongly adjust the hierarchy motif in the self‐assembly of azobenzene dyads to constitute discrete “unstackable” nanotoroids. We thus synthesized dyads **(*S*)‐2** and **2** bearing wedge‐shaped trialkoxybenzamide units (Figure 1 a,c). These new dyads self‐assemble into homogenous nanotoroids that, contrary to **(*S*)‐1**, are not able to interact together into tubular stacks due to the steric constraints imposed by the trialkoxybenzamide units. To our delight, atomic force microscopy (AFM) imaging reveals the ability of **(*S*)‐2** and **2** to organize into highly ordered 2D packing network on a graphite substrate through a MCH solution processing (Figure 1 c). Importantly, we demonstrate the strong influence of the solvent to achieve this hierarchical organization since this 2D hexagonal arrangement is not visualized neither in *n*‐octane nor in *n*‐dodecane. We also show that the photoisomerization of azobenzene moieties reversibly modulate the formation of the nanotoroids. The results presented herein represent an innovative strategy to bias the hierarchy motifs of supramolecular nanoelements to build up stimuli‐responsive self‐assembled materials.^[17]^

## Results and Discussion

Azobenzene dyads **(*S*)‐2** and **2** were prepared with *trans* configuration following the synthetic procedure shown in Scheme S1, being the last step the esterification of the dicarboxylic acid **3** with the corresponding 3,4,5‐trialkoxybenzamide derivatives **(*S*)‐4** or **4**. These moieties have been often employed to achieve highly soluble self‐assembling units capable to experience efficient and kinetically controlled supramolecular polymerizations.^[18]^

The self‐assembly of azobenzene dyads **(*S*)‐2** and **2** has been initially studied by IR and NMR experiments. The involvement of the amide groups in the operation of H‐bonding, and also the π‐stacking of the azobenzene units, is confirmed by variable temperature (VT) ^1^H NMR experiments by using MCH‐d_14_ as solvent. In these experiments, decreasing the temperature results in a broadening effect of all the resonances concomitant with the appreciable deshielding of the triplet corresponding to the amide proton resonance and the upfield shift of the aromatic proton signals due to the aggregation of the monomeric units (Figure S1).^[19]^ The IR spectra of both **(*S*)‐2** and **2** in MCH show the Amide I stretching band at ca. 1635 cm^−1^, implying the setting up of H‐bonding interactions between the amide functional groups (Figure S2). The N−H stretching band appears at ca. 3300 cm^−1^, a wavenumber comparable to that described for intermolecularly H‐bonded amides (Figure S2).^[20]^ Notably, in more polar chloroform, the IR and VT‐^1^H NMR experiments demonstrate the formation of a 7‐membered intramolecularly H‐bonded pseudocycle. In this solvent, two weak bands at ca. 3453 and ca. 3410 cm^−1^, ascribable to free and intramolecularly H‐bonded N−H groups, are observed (Figure S2).^[20]^ The ester carbonyl vibrational bands in CHCl_3_ appears at lower wavenumber compared to those in MCH/*n*‐dodecane, confirming they form intramolecular H‐bonds in CHCl_3_ while they are free in the nonpolar solvents (Figure S2). Furthermore, VT‐^1^H NMR experiments in CDCl_3_ (*c*_T_=100 μM) as solvent disclose no shifts of the aromatic resonances but a slight shielding of the triplet corresponding to the amide group (Figure S3). Since at these experimental conditions, azobenzene dyads **(*S*)‐2** and **2** are in a molecularly dissolved state, these shifts imply the formation of the intramolecularly H‐bonded pseudocycle.^[21]^

**Figure 2:**
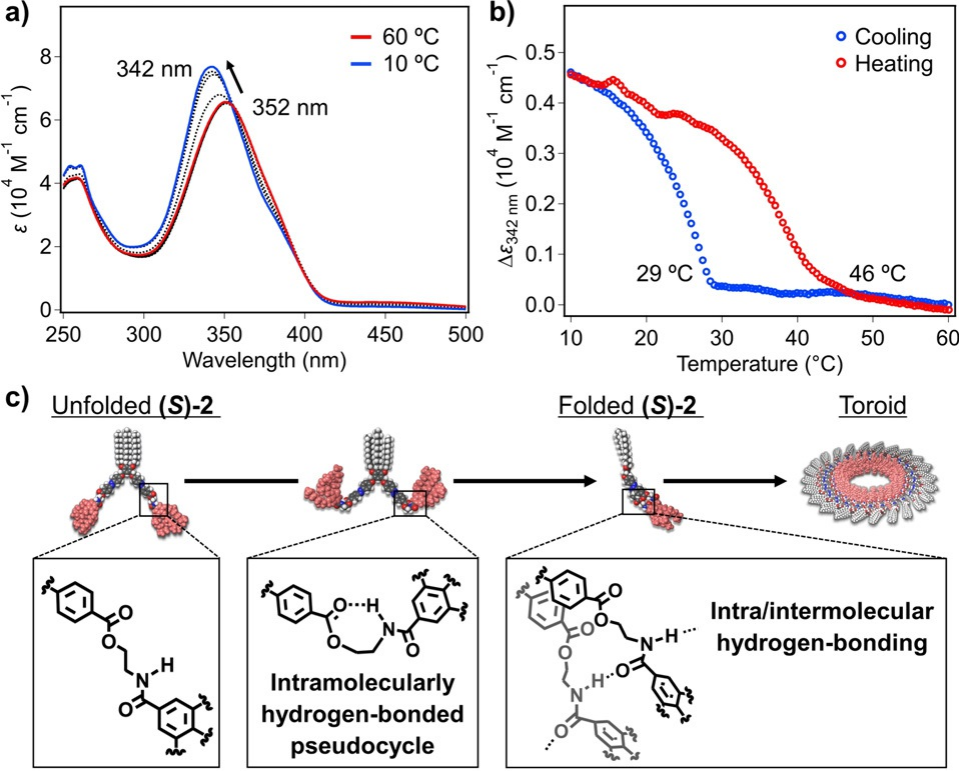
a) VT‐UV/Vis spectra of **(*S*)‐2** in MCH (*c*_T_=300 μM) at different temperatures. The temperature interval between spectra was 5 °C. b) Plots of the variation of the molar extinction coefficient (*ϵ*) of **(*S*)‐2** at 342 nm versus temperature extracted from VT‐UV/Vis spectra. c) Representations of the self‐assembly process of **(*S*)‐2**.

Self‐assembly of **(*S*)‐2** and **2** in non‐polar solvent was studied by using VT‐UV/Vis experiments. As previously described for azobenzene‐based supramolecular polymers,[^15^, ^22^] the face‐to‐face stacking is inferred from variable temperature VT‐UV/Vis spectra in MCH as solvent and at *c*_T_=300 μM, unravelling a red‐shift of the π–π* transition of the *trans*‐azobenzene moieties from *λ*_max_=352 to 342 nm upon cooling (Figure 2 a and S4a). Plotting the degree of aggregation (α), estimated from the variation of the absorbance at *λ*=342 nm, as a function of temperature results in non‐sigmoidal curves, which enables discarding an isodesmic mechanism for the supramolecular polymerization of both dyads.[^23^, ^24^] Thus, the two‐step plots display a smooth change in the degree of aggregation at high temperatures (333–305 K), and a sharp transition at an elongation temperature (*T*_e_) around 305 K that resembles to a nucleated supramolecular polymerization (Figure S5).^[24]^ These curves cannot be globally fitted to any of the previously models reported for cooperative processes, which has been justified by a prenucleation event ascribed to the conformational changes of the dyad. In fact, the folding of the azobenzene dyad through intramolecular π‐stacking to give wedge‐shaped building blocks that further grow to yield the final aggregates, could reasonably account for this prenucleation event. The elongation regime, observed in the lower temperature regime, can be fitted to the cooperative model described by Meijer and co‐workers. The preliminary results unveil the almost same stability of the aggregates formed by chiral **(*S*)‐2** (*ΔH*=−94.9 kJ mol^−1^; *T*_e_=302 K) in comparison to achiral **2** (*ΔH*=−98.0 kJ mol^−1^; *T*_e_=306 K) (Figure S5).^[25]^ Subsequent heating curves displayed significant hysteresis (*ΔT*_e_=17 °C for **(*S*)‐2**, *ΔT*_e_=20 °C for **2**, Figure 2 b and S4b). This dissimilarity between cooling and heating curves is diagnostic of the presence of an aggregation‐inactive conformation.[^20d^, ^20e^, ^21^] We attributed the observed thermal hysteresis to the transformation of the dyad from the open conformation featuring the intramolecularly H‐bonded pseudocycles to the folded one with intra/intermolecular hydrogen‐bonding, which could retard the nucleation upon cooling (Figure 2 c).

Since the hierarchical levels of stackable nanotoroids of **(*S*)‐1** into nanotubes could be recognized by the emergence of large circular dichroism (CD) activity,^[15a]^ we also studied self‐assembly of chiral **(*S*)‐2** by CD spectroscopy. At 20 °C, the MCH solution of **(*S*)‐2** disclosed a weak positively bisignate CD signal with the zero‐crossing point at 342 nm (Figure S6a). The Cotton effect was indicative for a chiral exciton coupling of the azobenzene units. Contrary to the stackable nanotoroids of **(*S*)‐1**, decreasing the temperature for **(*S*)‐2** to 0 °C did not reveal an enhancement of the dichroic response characteristic of the formation of chiral nanotubes.^[15a]^ In good agreement with this observation, dynamic light scattering (DLS) measurements of **(*S*)‐2** showed temperature‐independent hydrodynamic diameter (*D*_H_) of around 10 nm (Figure S6b). These results suggest that **(*S*)‐2** forms only one type of discrete assembly even at 0 °C.

**Figure 3:**
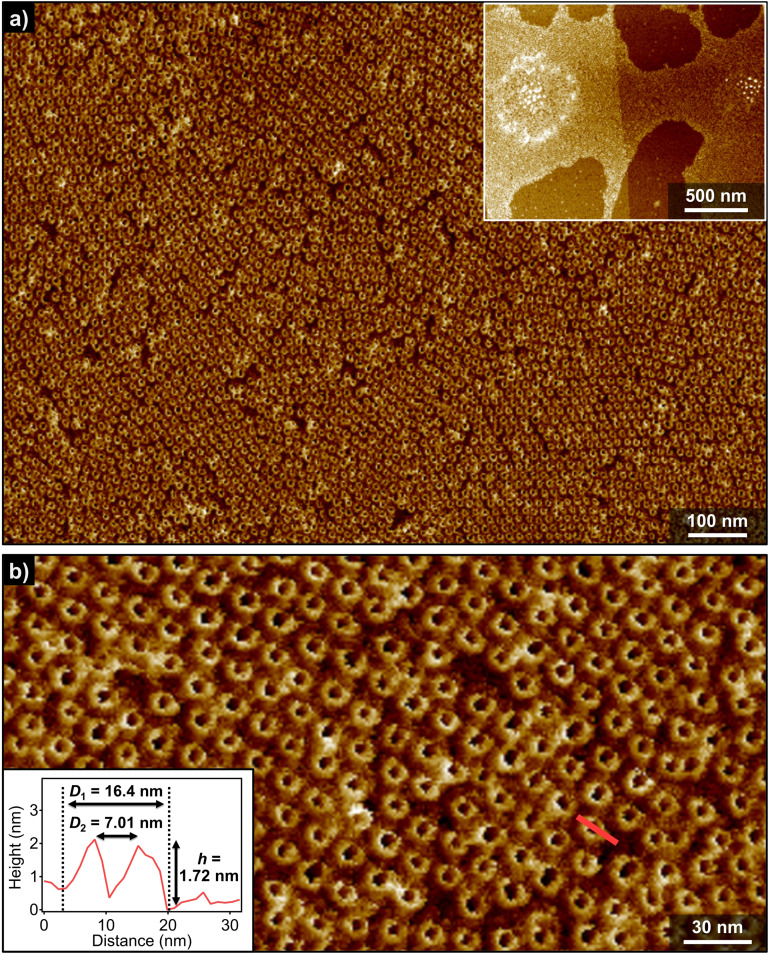
a),b) AFM images of nanotoroids of **(*S*)‐2** in MCH (*c*_T_=100 μM) obtained by cooling a hot MCH solution to 10 °C. Inset in (a) shows a wider range AFM image. Inset in (b) shows a cross‐sectional analysis along the pink line in (b).

We investigated the morphology of the aggregates formed by **(*S*)‐2** and **2** by AFM upon spin‐coating their solutions in MCH onto highly oriented pyrolytic graphite (HOPG) substrate. As representatively depicted in Figure 3, a massive number of toroidal structures was clearly visualized in a wide range of the specimen. These toroidal morphologies of **(*S*)‐2** and **2**, and their 2D hexagonal packing, were reproducibly observed regardless the conditions utilized to prepare the sample visualized for AFM (Figure S7 and S8). Importantly, unlike the previous azobenzene dyads, tubular aggregates were not detected even upon increasing the solute concentration, lowering the solution temperature, and aging the solutions for days. Combined with the CD and DLS results, these observations corroborate that toroidal assemblies of **(*S*)‐2** and **2** are discrete species, which can, however, form organized 2D networks. This 2D hexagonal packing formed by the toroids of **(*S*)‐2** and **2** illustrates outstanding potential of our scissor‐shaped molecular design leading to highly uniform and discrete nanostructures.^[16]^

**Figure 4:**
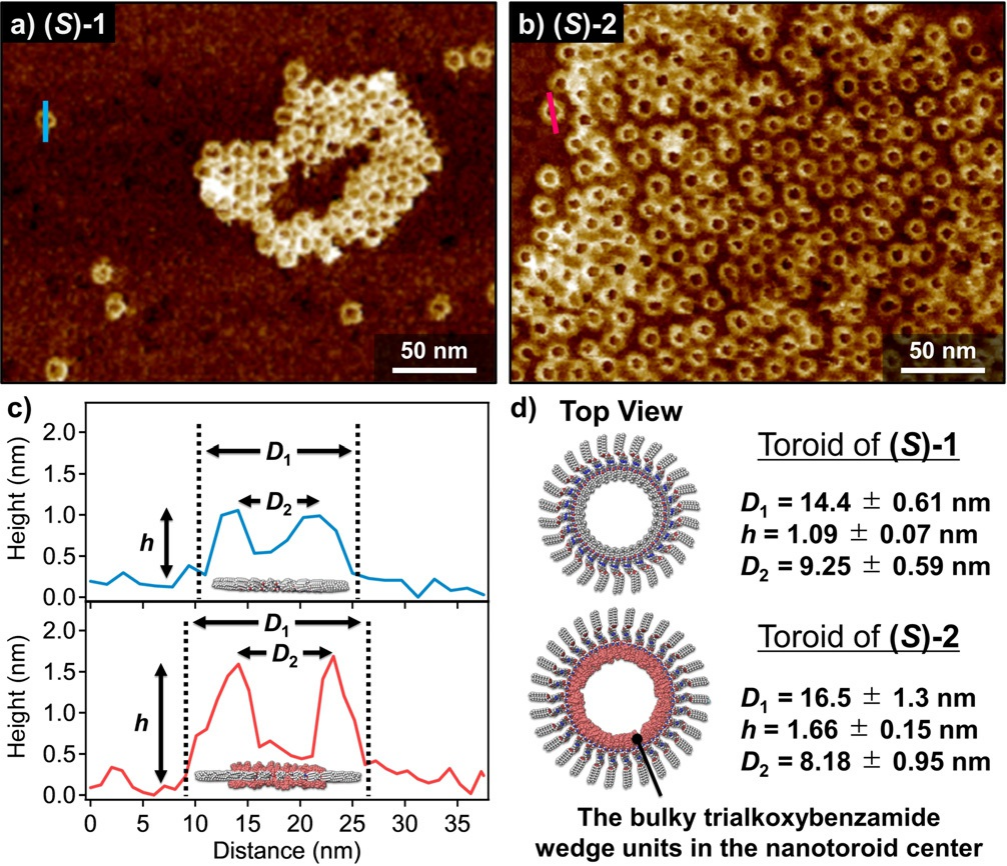
a),b) AFM images of a) stackable nanotoroids of **(*S*)‐1** and b) unstackable nanotoroids of **(*S*)‐2** obtained by spin‐coating of these MCH solutions onto HOPG substrates. c) AFM cross‐sectional analysis of the nanotoroids formed by **(*S*)‐1** (along the blue line in (a)) and by **(*S*)‐2** (along the pink line in (b)). d) Representations of the proposed toroidal structures of **(*S*)‐1** and **(*S*)‐2**, respectively. Bulky wedge groups located inside the nanotoroid of **(*S*)‐2**.

To further shed light into the morphological difference between the previous stackable nanotoroids of **(*S*)‐1** and the present unstackable nanotoroids formed by **(*S*)‐2**, AFM analysis of these structures was carefully performed using higher resolution AFM cantilever (Figure 4, see the Supporting Information). For previous stackable nanotoroids of **(*S*)‐1**, edge‐to‐edge diameter (*D*_1_), top‐to‐top diameter (*D*_2_), and height (*h*) were estimated as *D*_1_=14.4±0.61 nm, *D*_2_=9.25±0.59 nm, and *h*=1.09±0.07 nm, respectively (Figure 4 a,c,d). For unstackable nanotoroids of **(*S*)‐2**, these dimensions were estimated as *D*_1_=16.5±1.3 nm, *D*_2_=8.18±0.95 nm, and *h*=1.66±0.15 nm, respectively (Figure 4 b–d). The larger *h* value of **(*S*)‐2** clearly reflects the presence of bulky trialkoxybenzamide wedge units. Interestingly, and despite the fact that *D*_1_ of **(*S*)‐2** is larger than that of **(*S*)‐1**, *D*_2_ of **(*S*)‐2** was measured shorter than that of **(*S*)‐1**. As schematically shown in Figure 4 c and 4 d, the above counterintuitive values of *D*_2_ strongly suggests that **(*S*)‐2** self‐assembles in a similar manner to that reported for **(*S*)‐1**, i.e., directing the tri(dodecyloxy)xylylene linker outside whereas the bulky wedge units, which point to the inner pore of the toroidal structure (Figure 4 d). Noteworthy, the nanotoroids of achiral **2** have almost same dimensions with those of **(*S*)‐2**, implying that the branched alkyl chains scarcely affect the morphology of the aggregated nanostructures (Figure S9).

The above‐mentioned AFM cross‐sectional analysis suggests that the molecular packing structure of both stackable and unstackable nanotoroids is almost identical, i.e., the outer and inner parts of the nanotoroids are wrapped by 3,4,5‐tri(dodecyloxy)benzene (xylylene) moieties and wedge‐shaped side chains, respectively. However, the structural differences between **(*S*)‐1** and **(*S*)‐2** (or **2**) strongly affect their hierarchical self‐assembly. Since the toroidal nanostructure of **(*S*)‐1** shows a planar discotic geometry, these aggregates readily stack into a columnar assembly. In stark contrast, the nanotoroids formed by **(*S*)‐2** and **2** are relatively rugged owing to the bulky aliphatic wedges. This steric hindrance, imposed by the inner segment of these nanotoroids, prevents the hierarchical stacking into nanotubes, leading to discrete nanotoroids that can further interact through van der Waals contacts between the dodecyl chains of the tri(dodecyloxy)benzene (xylylene) moiety to generate the final 2D porous networks (Figure 1 c).

**Figure 5:**
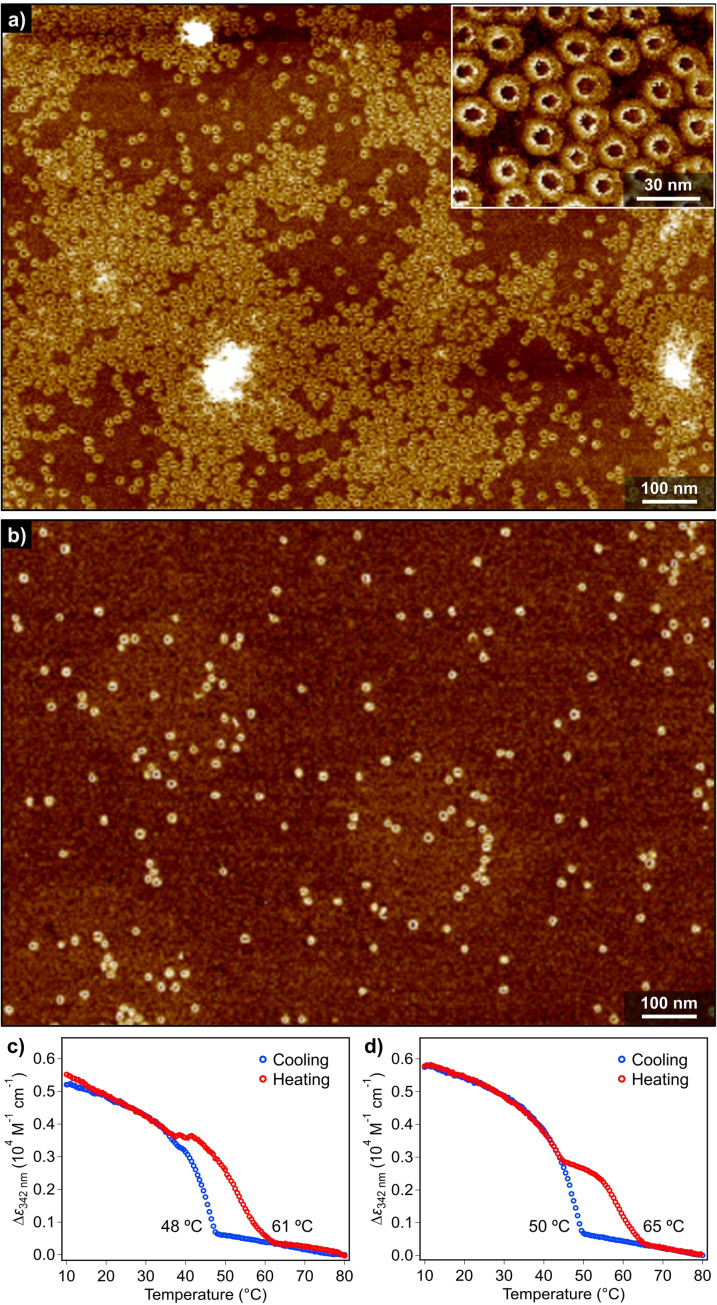
a),b) AFM image of nanotoroids of **(*S*)‐2** in a) *n*‐octane and b) *n*‐dodecane (*c*_T_=100 μM) obtained by cooling a hot MCH solution to 10 °C. Inset in (a) shows a magnified AFM image of nanotoroids. c),d) Plots of the variation of the molar extinction coefficient (*ϵ*) of **(*S*)‐2** at 342 nm versus temperature in c) *n*‐octane and d) *n*‐dodecane extracted from VT‐UV/Vis spectra.

It is well‐established that solvent is a key factor conditioning the length and, therefore, the function of supramolecular polymers due to the dynamic character of the non‐covalent interactions that join together the constitutive self‐assembling units.^[26]^ For simple one‐dimensional supramolecular polymers, use of lesser polar solvents generally increase degree of aggregation and enhance interaction between the resulting aggregates. However, for the present nanotoroids of **(*S*)‐2** and **2**, an opposite tendency has been observed. Figure 5 a and 5 b (and also see Figure S10) shows the AFM images of spin‐coated films of **(*S*)‐2** dissolved in *n*‐octane and *n*‐dodecane, respectively, at the same concentration (*c*_T_ *=*100 μM).

For the film prepared from the *n*‐octane solution, a similar amount of nanotoroids were observed as in the case of MCH. However, the nanotoroids formed in *n*‐octane are less organized, giving rise to “islands” with heights higher than those of nanotoroids by 4–6 nm in places (Figure S10c,d). This difference is ascribable to the volatility of solvents, as more volatile MCH allows a more efficient kinetic adsorption of nanotoroids onto HOPG (see the discussion in the caption of Figure S10). In contrast, for the film prepared from the *n*‐dodecane solution, considerably lesser amount of nanotoroids was visualized compared to MCH and *n*‐octane. Micrometer‐scale imaging of the spin‐coated films did not show the formation of any island of nanotoroids (Figure S10e,f), which demonstrates that inherently low yield of nanotoroids in *n*‐dodecane. The same trend was observed for achiral **2**. Reflecting these findings, temperature‐dependent UV/Vis analysis of both **(*S*)‐2** and **2** in *n*‐dodecane (*c*_T_=300 μM) revealed the formation of two type of aggregates (Figure 5 d, S11 and S12). The one dissociating at lower temperature did not show thermal hysteresis, but that dissociating at higher temperature did. The latter thermally stable species can be attributed to nanotoroids. For the former species, we infer that, in *n*‐dodecane, the intramolecularly hydrogen‐bonded dyads (Figure 2 c) are more stable due to over‐enhanced intramolecular interactions and cannot self‐assemble into nanotoroids. Such dormant monomers could weakly aggregate only through π‐stacking interaction to show reversible absorption change below 50 °C without hysteresis. This is supported by the presence of molecular‐level thin assemblies (0.77 nm in thickness) observed especially for achiral **2** only in *n*‐dodecane (inset in Figure S12d). These thin assemblies can be attributed to substrate‐templated assemblies of **2** bearing nine linear dodecyl chains which can epitaxially orient along to the crystal lattice of graphite.

Finally, we explored the photoresponsive properties of **(*S*)‐2** in monomeric and self‐assembled states.[^10^, ^27^] When a monomeric solution of **(*S*)‐2** in CHCl_3_ (*c*_T_=300 μM) was irradiated with UV light (*λ*=365 nm), a smooth *trans*→*cis* isomerization of the azobenzene moieties was confirmed by an attenuation of the π–π* absorption band at *λ*=363 nm and the growth of a new band at *λ*=446 nm (Figure S13d). At a photostationary state (PSS_UV_), the *trans*:*cis* ratio of azobenzene moieties was estimated to be 4:96 by ^1^H NMR spectroscopy analysis, from which the molar ratio of the three possible photoisomers, *trans*,*trans*‐ (*tt*‐), *trans*,*cis*‐ (*tc*‐) and *cis*,*cis*‐ (*cc*‐) of compound **(*S*)‐2**, was determined to be *tt*‐**(*S*)‐2**:*tc*‐**(*S*)‐2**:*cc*‐**(*S*)‐2**=1:5:94 (Figure S13). Upon visible‐light irradiation (*λ*=470 nm) of the PSS_UV_ solution, a smooth *cis*→*trans* back‐isomerization was confirmed by the recovery of π–π* absorption band (Figure S13d), resulting in another photostationary state (PSS_Vis_) at which the *trans*:*cis* ratio was estimated to be 78:22. The ^1^H NMR spectrum of the PSS_Vis_ state suggests that the ratio of the photoisomer is *tt*‐**(*S*)‐2**:*tc*‐**(*S*)‐2**:*cc*‐**(*S*)‐2**=71:23:6 (Figure S13b and S13c), which is in good accordance with the result of absorption measurement of the PSS_Vis_ solution.

**Figure 6:**
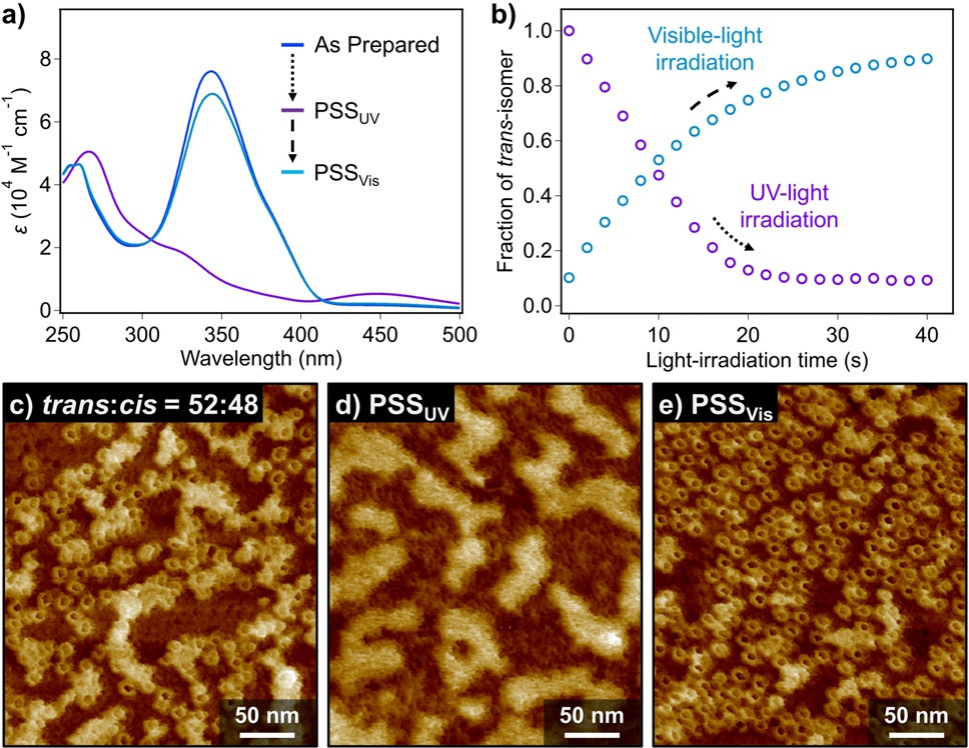
a) UV/Vis spectra of **(*S*)‐2** in MCH (*c*_T_=300 μM) at 20 °C recorded at as‐prepared (blue spectrum), PSS_UV_ (purple spectrum) and PSS_Vis_ (sky‐blue spectrum), respectively. b) Plots of the change in the mole fraction of *trans*‐isomer as a function of UV‐ (purple circles) and visible‐light (sky‐blue circles) irradiation time, respectively. c)–e) AFM images of **(*S*)‐2** in MCH (*c*_T_=300 μM) obtained by irradiation with c),d) UV‐ and e) visible light to the as‐prepared MCH solution. These solutions were diluted to 100 μM before spin‐coating onto HOPG substrates.

The *trans*→*cis* photoisomerization of **(*S*)‐2** smoothly proceeded also in the self‐assembled state in MCH (*c*_T_=300 μM) as shown by absorption measurements upon UV‐light irradiation (Figure 6 a). The corresponding PSS_UV_ was achieved by UV‐irradiation for 40 s, at which the *trans*:*cis* ratio was determined to be 9:91 (Figure 6 b). AFM observation for a partially photoisomerized sample (*trans*:*cis*=52:48) revealed the ill‐defined films covering nanotoroids (Figure 6 c). At the PSS_UV_, the nanotoroids of *tt*‐**(*S*)‐2** completely disappeared and only ill‐defined films with a height of ca. 2.1 nm were observed (Figure 6 d, S14a and S14b). This result suggests that photogenerated *cis*‐containing isomers, i.e., *tc*‐ and *cc*‐**(*S*)‐2** cannot form well‐defined nanostructures.

Subsequent irradiation of the PSS_UV_ solution of **(*S*)‐2** with visible light (*λ*=470 nm) resulted in an increase of the π–π* absorption band of *trans*‐azobenzene units, leading to a PSS_Vis_ with the *trans*:*cis* ratio of 90:10 upon 40 s irradiation (Figure 6 a,b). AFM imaging of a sample prepared by spin‐coating of the PSS_Vis_ solution revealed a substantial recovery of pristine nanotoroids (Figure 6 e, S14c and S14d). When the PSS_UV_ and PSS_Vis_ were repeatedly generated by mutual irradiation with UV and visible light, respectively, reversible transition of average *D*_H_ between ca. 9 nm and 1 nm was confirmed at least 3 times by DLS measurements (Figure S15 and S16). This demonstrates the photoreversibility between the toroidal and molecularly dissolved states.

To further understand the relationship between the photoisomerization and the dissociation of the nanotoroids, we investigated the transition of the *trans*/*cis* isomer ratio of azobenzene moieties until the PSS_UV_ was achieved. Interestingly, the fraction of the *trans*‐isomer decreased linearly by UV‐light irradiation, which is very different from its mono‐exponential increase by visible‐light irradiation (Figure 6 b). The difference is probably due to the formation of nanotoroids by *tt*‐**(*S*)‐2** in the former process, while in the latter process *cc*‐**(*S*)‐2** does not form well‐defined aggregate. Indeed, when UV‐light irradiation was carried out for *tt*‐**(*S*)‐2** at a sufficiently low concentration (*c*_T_=40 μM) wherein no toroidal aggregates were detected by AFM, mono‐exponential decrease of the fraction of the *trans*‐isomer was observed (Figure S17). We thus infer that the *trans*→*cis* photoisomerization of *tt*‐**(*S*)‐2** is assumed to be kinetically regulated by nanotoroid‐monomer equilibrium. Namely, only free *tt*‐**(*S*)‐2** can photoisomerize whereas those in nanotoroids cannot. Once free *tt*‐**(*S*)‐2** partially photoisomerize into *tc*‐**(*S*)‐2**, this partially crippled molecule must be incapable of aggregating, and accordingly smoothly isomerize into *cc*‐**(*S*)‐2**.

## Conclusion

We present scissor‐shaped azobenzene dyads **(*S*)‐2** and **2** bearing sterically demanding 3,4,5‐tridodecyloxy benzamide moieties at the end of azobenzene bodies through a flexible linker. These new azobenzene dyads are capable of constituting discrete nanotoroids in apolar solvents like MCH, *n*‐octane and *n*‐dodecane. Importantly, the steric demand in the nanotoroid interior exerted by the trialkoxybenzamide units results in the interaction between the outer peripheral side chains, thereby changing their hierarchical organization architectures from 1D to 2D. Noteworthy, the characteristics of the solvent plays a relevant role on the hierarchical organization and the yield of the nanotoroids. Thus, whilst in the more volatile MCH a highly organized 2D hexagonal arrangement of the nanotoroids is observed, this 2D hexagonal organization is not observed in less volatile *n*‐octane. Interestingly, in the less volatile *n*‐dodecane, however, a reduced number of nanotoroids is observed, most probably due to the higher stability of the intramolecularly H‐bonded pseudocycle that prevents the efficient formation of the nanotoroids. These nanotoroids dispersed in solution showed efficient photoresponsive dissociation and reorganization due to the *trans*↔*cis* isomerization of azobenzene moieties. Although this photoresponsive property is currently limited to solution phase, we are currently investigating the possibility of the same photoresponsive property at the air‐liquid interface to develop photoresponsive porous nanosheets.

## Conflict of interest

The authors declare no conflict of interest.

## Supporting information

As a service to our authors and readers, this journal provides supporting information supplied by the authors. Such materials are peer reviewed and may be re‐organized for online delivery, but are not copy‐edited or typeset. Technical support issues arising from supporting information (other than missing files) should be addressed to the authors.

Supporting Information
