# Supplementary material for: Biasing the Hierarchy Motifs of Nanotoroids: from 1D Nanotubes to 2D Porous Networks
Source: Angew Chem Int Ed Engl. 2021 Dec 15;61(5):e202114290. doi: 10.1002/anie.202114290 (PMC9299728; doi:10.1002/anie.202114290)
Supplement: Supplementary file 1 — Supporting Information [file ANIE-61-0-s001.pdf]

Angewandte Chemie
Eine Zeitschrift der Gesellschaft Deutscher Chemiker

Supporting Information

Biasing the Hierarchy Motifs of Nanotoroids: from 1D Nanotubes to 2D Porous Networks

Jorge S. Valera[+], Hironari Arima[+], Cristina Naranjo, Takuho Saito, Natsuki Suda, Rafael Gómez, Shiki Yagai, and Luis Sánchez**

anie_202114290_sm_miscellaneous_information.pdf

Table of Contents

1.- Material and Methods S-3

2.- Synthesis and Characterization S-3

3.- Supplementary Figures S-7

VT-^{1}H NMR experiments in MCH-d14 S-7

FTIR spectra in solution S-8

VT-^{1}H NMR experiments in $CDCl_3$ S-8

VT-UV-Vis experiments in MCH S-9

VT-CD experiments and DLS measurements in MCH S-9

AFM images in MCH, octane and dodecane S-10

VT-UV-Vis experiments S-12

Photoirradiation experiments (UV-Vis, DLS, AFM) S-13

4.- References S-16

1- Materials and Methods

Materials: All commercially available reagents and solvents were of reagent grade and used without further purification. The solvents for the preparation of the assemblies were all spectral grade and used without further purification. Column chromatography was performed using 63–210 μm silica gel. Preparative gel permeation chromatography (GPC) was performed on a recycling preparative HPLC (LC-9225NEXT, Japan Analytical Industry) equipped with GPC columns JAIGEL-1H+2H). ^{1}H and ^{13}C NMR spectra were recorded on Bruker-AVANCEIII-400M (400 MHz) NMR spectrometer. VT-^{1}H NMR spectra were recorded on a Bruker Avance 300 (300 MHz) NMR spectrometer. ^{1}H NMR chemical shifts reported in parts per million (ppm, δ) were referenced to the chemical shifts of tetramethylsilane (TMS) at 0.00 ppm. The resonance multiplicity is described as s (singlet), d (doublet), t (triplet), dd (double doublet), m (multiplet) and br (broad). ^{13}C NMR chemical shifts reported in parts per million (ppm, δ) were referenced to the chemical shifts of $CDCl_3$ at 77.16 ppm. APCI-MS spectra were measured on an Exactive (Thermo Scientific).

UV-Vis and circular dichroism (CD) spectroscopy: UV-Vis absorption spectra were recorded on a JASCO V660 or JASCO V760 spectrophotometer equipped with JASCO ETCS-761 temperature controller. CD spectra were recorded on a JASCO J840 spectropolarimeter equipped with JASCO PTC-423L temperature controller. These spectra were recorded by using a screw capped quartz cuvettes with an optical pathlength of 1.0 mm.

DLS measurement: DLS measurements were performed on a Zetasizer Nano S (Malvern Instruments) device using non-invasive back-scatter technology (NIBS) under 4.0 mW He-Ne laser (633 nm). The scattering angle was set at 173°. Temperature-dependent viscosities and refractive indexes were added manually.

Fourier transform Infrared (FT-IR) measurement: FT-IR spectra were measured on JASCO FT/IR-4600 spectrometer by using a KBr cell with an optical pathlength of 1.0 mm.

Atomic force microscopy (AFM): AFM images were acquired under ambient conditions using Multimode 8 Nanoscope V microscope (Bruker Instruments) in peak force tapping (ScanAsyst) mode. Silicon cantilevers (SCANASYST-AIR) with a spring constant of 0.4 N m^{-1} and frequency of 70 kHz (nominal value, Bruker, Japan) were used. AFM images were acquired under ambient conditions using Multimode 8 Nanoscope V microscope (Bruker Instruments) in peak force tapping (ScanAsyst) mode. Silicon cantilevers (SCANASYST-AIR-HPI) with a spring constant of 0.25 N m^{-1} and frequency of 55 kHz (nominal value, Bruker, Japan) were used. Samples were prepared by spin-coating (3,000 rpm, 1 min) assembly solutions onto freshly cleaved highly-oriented pyrolytic graphite (HOPG, 5 × 5 mm^2).

Calculation of α: Degree of aggregation (α) was calculated from molar extinction coefficient (ε) at 342 nm for **(*S*)-2** and **2** in VT-UV-Vis spectra, based on following equation:

$$\alpha = \frac{\varepsilon_{max} - \varepsilon}{\varepsilon_{max} - \varepsilon_{min}}$$

wherein ε_{max} is the molar extinction coefficient of fully aggregated state estimated from the absorption spectra at 10 °C (α = 1), and ε_{min} is the molar extinction coefficient of fully monomeric state estimated from the absorption spectra at 60 °C (α = 0).

Photoirradiation experiments: The samples were irradiated by using LED lamps (For UV-light irradiation: λ = 365 nm, 17 mW cm^{-2}, at a distance of 5 cm, for visible-light irradiation: λ = 470 nm, 33 mW cm^{-2}, at a distance of 5 cm)

2.- Synthesis and Characterization

Compound **(*S*)-2** and **2** were synthesized according to Scheme S1. Compounds **3**[S1], **(*S*)-4**[S2] and **4**[S2] were synthesized according to the procedures reported previously.

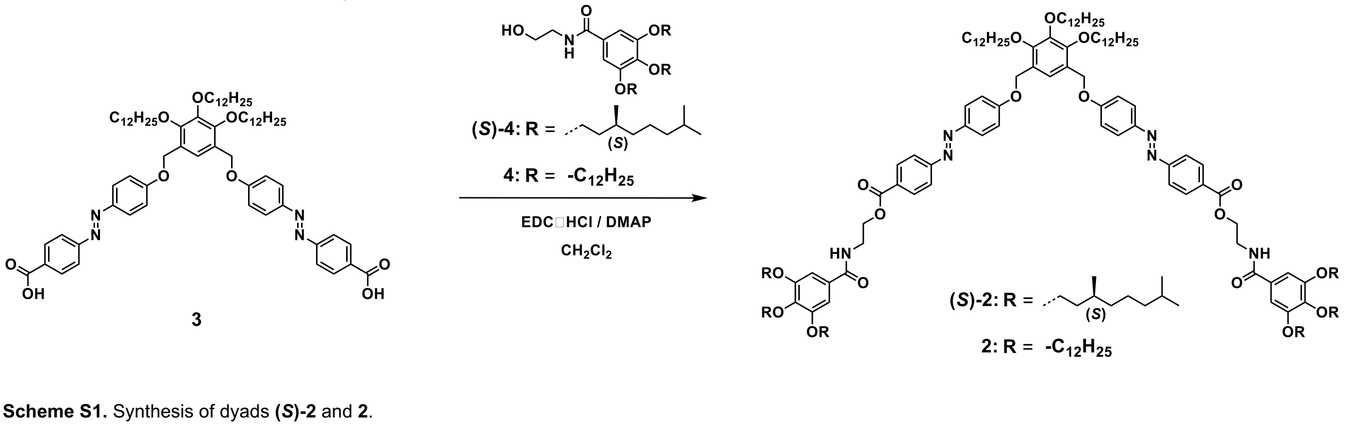

Scheme S1. Synthesis of dyads **(*S*)-2** and **2**.

Compound (*S*)-2: Compound **3** (140 mg, 0.123 mmol), 1-(3-dimethylaminopropyl)-3-ethylcarbodiimide hydrochloride (EDC·HCl, 118 mg, 0.616 mmol) and *N*,*N*-dimethylpyridin-4-amine (DMAP, 31 mg, 0.25 mmol) were dispersed in 5 mL of dry CH_2Cl_2 at r.t. and the mixture was sonicated. To this mixture, compound **(*S*)-4** (160 mg, 0.25 mmol) in 2 mL of dry CH_2Cl_2 was added portionwise and the mixture was stirred for 18 h at r.t. The reaction mixture was washed with brine. The organic layer was dried over Na_2SO_4 and evaporated *in vacuo*. The residue was purified by column chromatography over silica gel (eluent: $CHCl_3$/MeOH = 200/1; v/v%) and further purified by gel permeation chromatography (GPC, eluent: $CHCl_3$) to give **(*S*)-2** as a waxy orange solid (60 mg, 21% yield).

^{1}H NMR (400 MHz, $CDCl_3$, 20 °C): δ = 8.12 (dd, *J* = 1.9, 8.7 Hz, 4H), 7.90 (dd, *J* = 1.9, 9.0 Hz, 4H), 7.85 (dd, *J* = 1.8, 8.7 Hz, 4H), 7.26 (s, 1H), 7.05 (dd, *J* = 2.0, 9.1 Hz, 4H), 7.02 (s, 4H), 6.74 (brt, *J* = 5.4 Hz, 2H), 5.14 (s, 4H), 4.59 (t, *J* = 5.1 Hz, 4H), 4.10 (t, *J* = 6.7 Hz, 4H), 4.05–3.96 (m, 14H), 3.88–3.87 (m, 4H), 1.88–1.11 (m, 120H), 0.92–0.83 (m, 63H).

^{13}C NMR (100 MHz, $CDCl_3$, 20 °C): δ = 167.69, 166.75, 161.90, 155.50, 153.12, 152.00, 146.98, 145.81, 140.99, 130.68, 130.48, 129.15, 125.26, 125.21, 122.47, 115.04, 105.41, 74.29, 73.78, 71.70, 67.48, 65.55, 64.01, 39.90. 39.36, 39.26, 37.51, 37.35, 37.31, 36.32, 31.98, 31.95, 30.43, 30.41, 29.81, 29.78, 29.74, 29.73, 29.69, 29.62, 29.56, 29.43, 29.41, 28.00, 26.21, 24.76, 24.74, 22.74, 22.72, 22.65, 22.64, 19.56, 14.17.

HRMS (APCI, m/z): [M+H] calcd for $C_{148}H_{237}O_{17}N_6$, 2370.7860, found 2370.7820.

Compound 2: Compound **2** was synthesized according to the abovementioned procedure for **(*S*)-2** from compound **3** (77 mg, 0.068 mmol), EDC·HCl (63 mg, 0.33 mmol), DMAP (16 mg, 0.13 mmol) and compound **4** (174 mg, 0.24 mmol) and obtained in 15% yield (waxy orange solid).

^{1}H NMR (400 MHz, $CDCl_3$, 20 °C): δ = 8.12 (dd, *J* = 1.9, 8.7 Hz, 4H), 7.90 (dd, *J* = 2.1, 9.0 Hz, 4H), 7.84 (dd, *J* = 1.9, 8.8 Hz, 4H), 7.25 (s, 1H), 7.05 (dd, *J* = 2.1, 9.1 Hz, 4H), 7.00 (s, 4H), 6.70 (brt, *J* = 5.4 Hz, 2H), 5.14 (s, 4H), 4.59 (t, *J* = 5.2 Hz, 4H), 4.10 (t, *J* = 6.7 Hz, 4H), 4.05–3.96 (m, 14H), 3.88–3.86 (m, 4H), 1.82–1.70 (m, 18H), 1.45–1.21 (m, 162H), 0.89–0.83 (m, 27H).

^{13}C NMR (100 MHz, $CDCl_3$, 20 °C): δ = 167.68, 166.72, 166.66, 161.90, 155.51, 153.10, 151.96, 147.00, 145.79, 141.73, 141.09, 130.67, 130.50, 129.14, 125.25, 125.21, 122.45, 122.40, 115.04, 105.54, 74.27, 73.50, 69.23, 65.55, 63.99, 39.88, 31.96, 30.49, 30.43, 30.40, 30.32, 29.98, 29.77, 29.75, 29.73, 29.67, 29.61, 29.55, 29.43 29.40, 29.35, 26.21, 26.10, 22.72, 14.16, 13.56.

HRMS (APCI, m/z): [M+H] calcd for $C_{160}H_{261}O_{17}N_6$, 2538.9738, found 2538.9692.

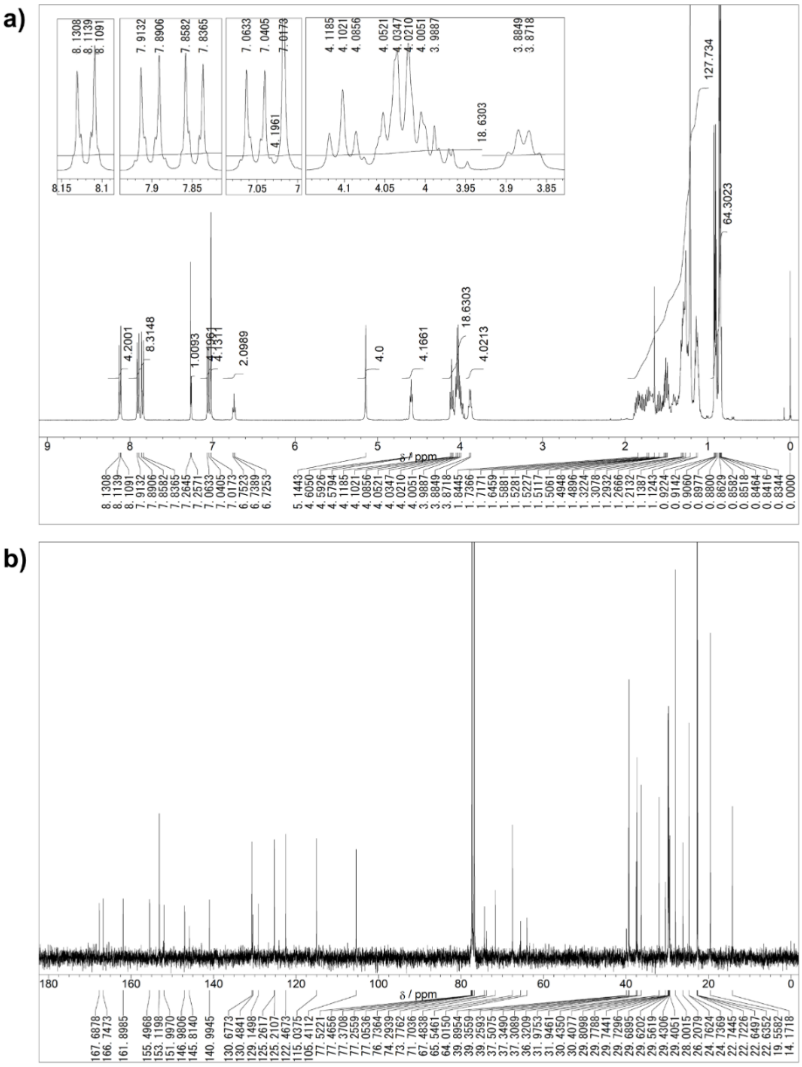

Chart S1. a) ^{1}H and b) ^{13}C NMR spectra of **(*S*)-2** in $CDCl_3$ at 20 ºC.

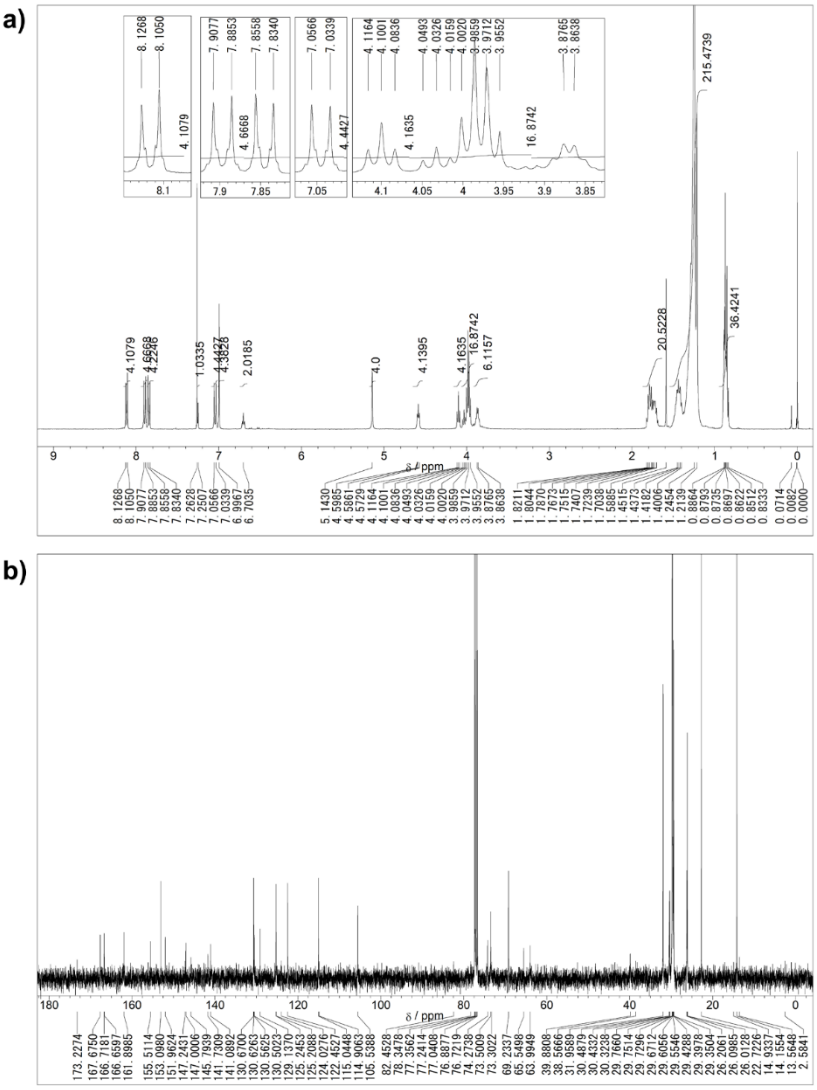

Chart S2. a) ^{1}H and b) ^{13}C NMR spectra of **2** in $CDCl_3$ at 20 ºC.

3.- Supplementary Figures

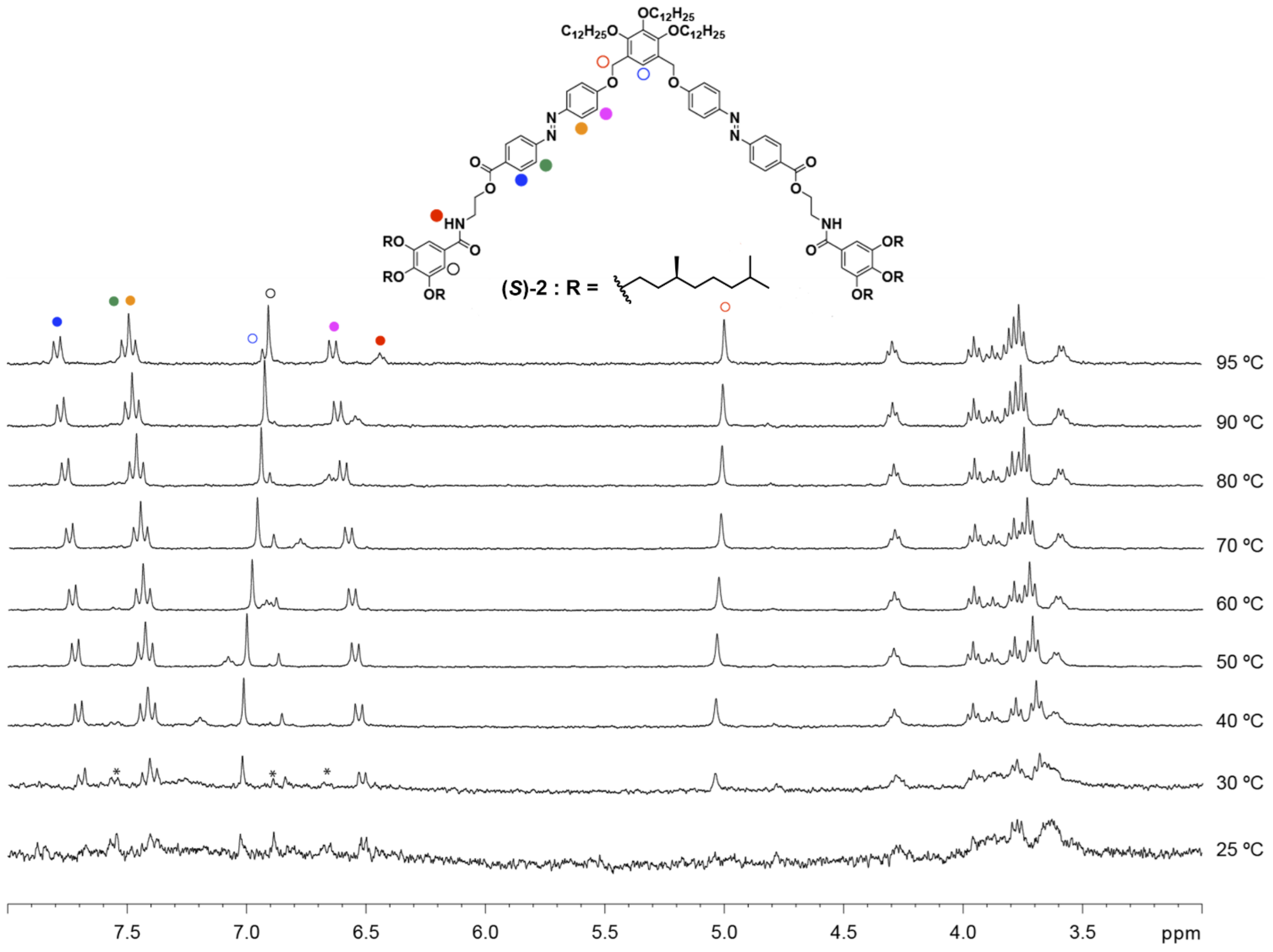

Figure S1. Partial ^{1}H NMR spectra of **(*S*)-2** (300 MHz; MCH-d$_{14}$; c_T = 100 μM). The asterisks indicate the resonances corresponding to the residual *cis*-isomers.

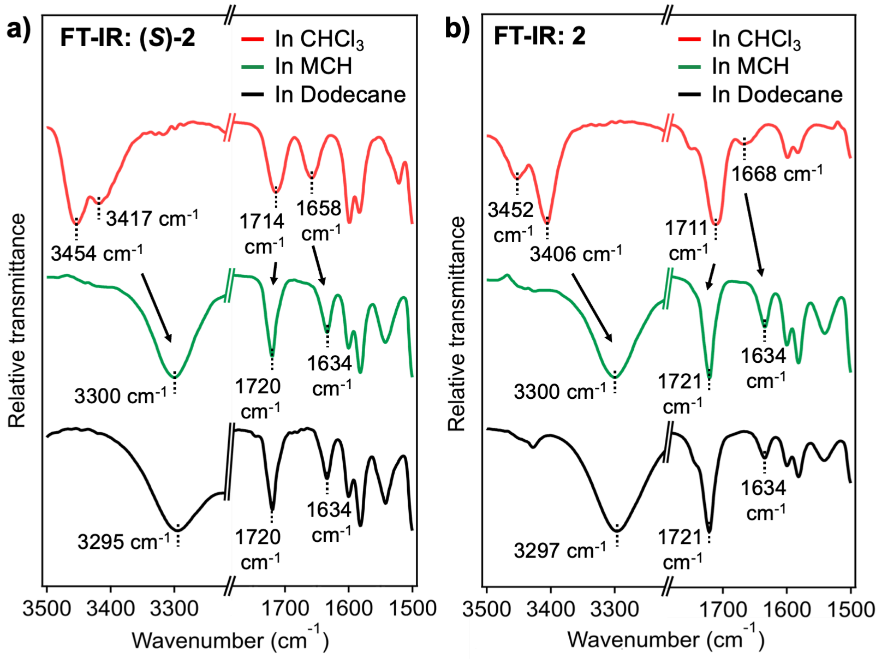

Figure S2. a,b) Partial FTIR spectra (N-H, ester carbonyl vibrational bands and Amide I bands) of (a) **(*S*)-2** and (b) **2** in $CHCl_3$ (c_T = 1 mM, red lines), MCH (c_T = 1 mM, green lines) and dodecane (c_T = 1 mM, black lines), respectively, recorded at 20 ºC.

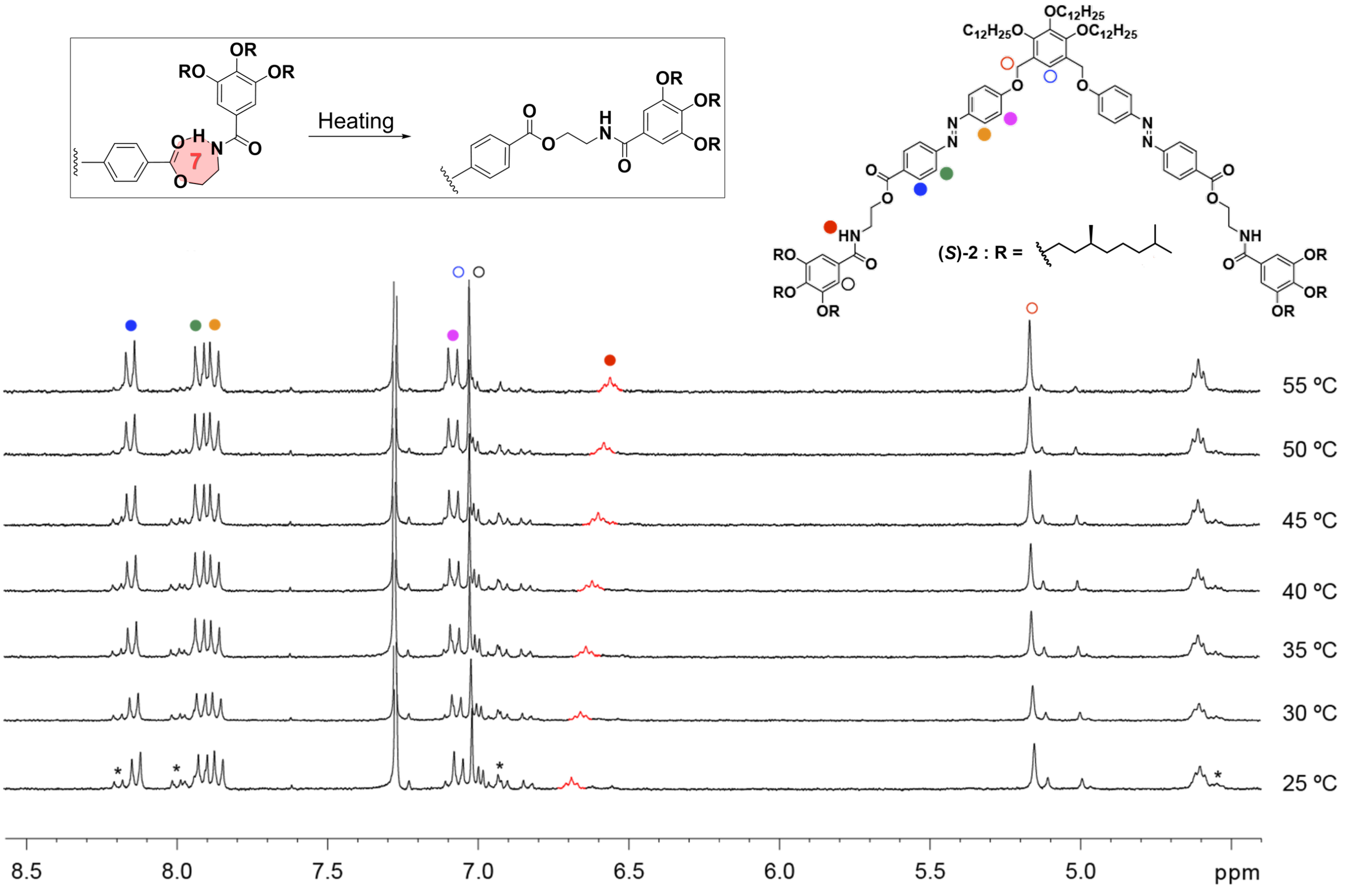

Figure S3. Partial ^{1}H NMR spectra of **(*S*)-2** at different temperatures (300 MHz; $CDCl_3$, c_T = 100 μM). The asterisks display the resonances corresponding to the *cis*-isomers. The upper part of the panel shows the rupture of the intramolecularly H-bonded pseudocycle upon heating.

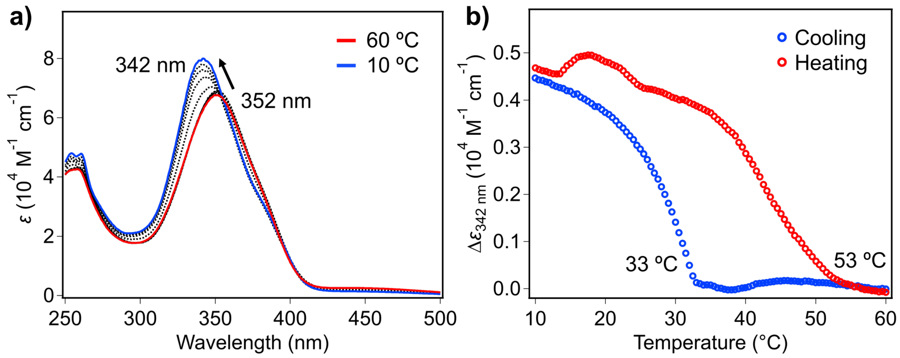

Figure S4. a) VT-UV-Vis spectra of **2** in MCH (c_T = 300 μM) upon cooling from 60 to 10 ºC at a rate of 1 ºC min^{-1}. The temperature interval between spectra was 5 ºC. Arrows indicate the changes upon decreasing temperature. b) Plot of the variation of the ε at 342 nm versus temperature extracted from VT-UV-Vis spectra of **2**.

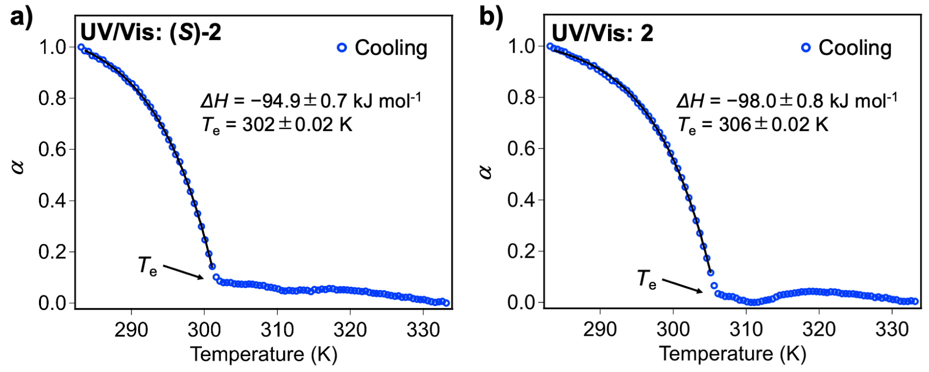

Figure S5. a,b) Temperature-dependent degree of aggregation (α) at 342 nm of (a) **(*S*)-2** and (b) **2** in MCH (c_T = 300 μM), respectively. The black solid curves were obtained by fitting the experimental data to the cooperative (nucleation–elongation) model proposed by Meijer and co-workers.[S3]

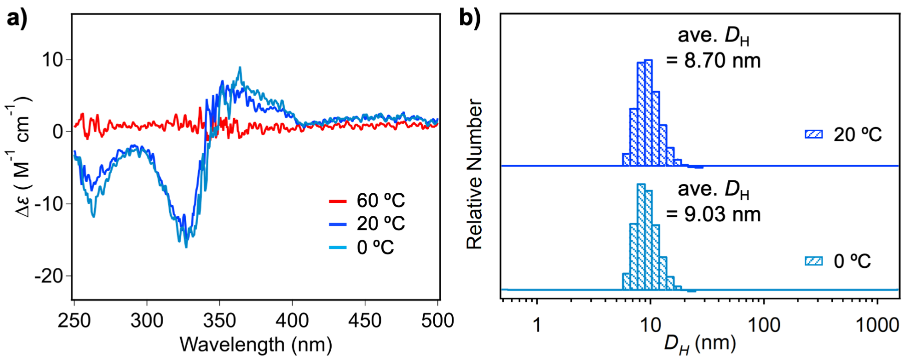

Figure S6. a) VT-CD spectra of **(*S*)-2** in MCH (c_T = 300 μM) at 60 (red spectrum), 20 (blue spectrum) and 0 ºC (sky-blue spectrum), respectively. b) DLS size distributions of **(*S*)-2** in MCH (c_T = 300 μM) at 20 ºC (blue bars) and 0 ºC (sky-blue bars), respectively.

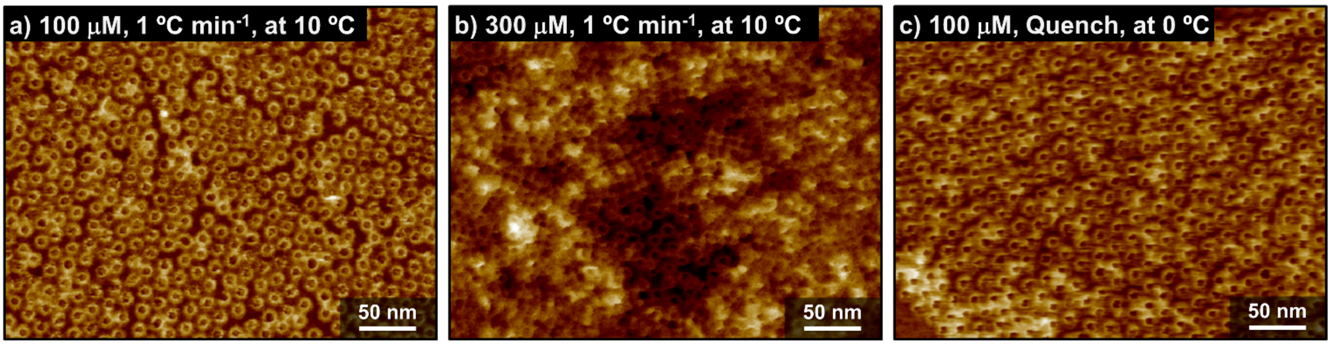

Figure S7. a–c) AFM images of nanotoroids of **(*S*)-2** in MCH. The samples were prepared by following conditions; (a): c_T = 100 μM, cooling at 1 °C min^{-1}, spin-coated at 10 °C; (b): c_T = 300 μM, cooling at 1 °C min^{-1}, spin-coated at 10 °C; (c): c_T = 100 μM, fast cooling (quench), spin-coated at 0 °C.

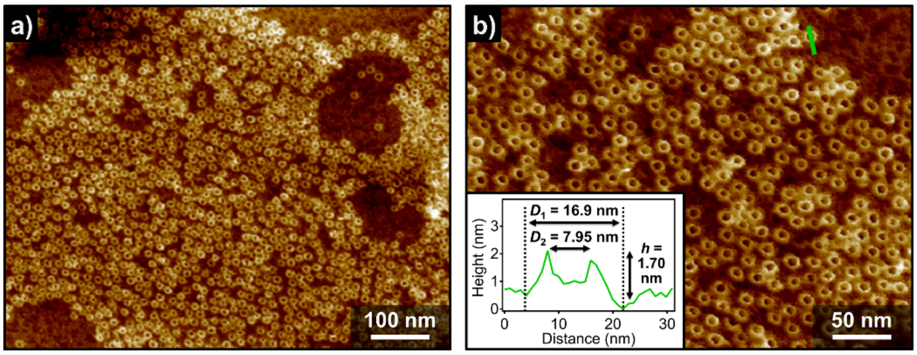

Figure S8. a,b) AFM images of nanotoroids of **2** in MCH (c_T = 100 μM) obtained by cooling a hot solution to 0 °C at a rate of 1 °C min^{-1}. Inset in (b) shows a cross-sectional analysis along the green line in (b).

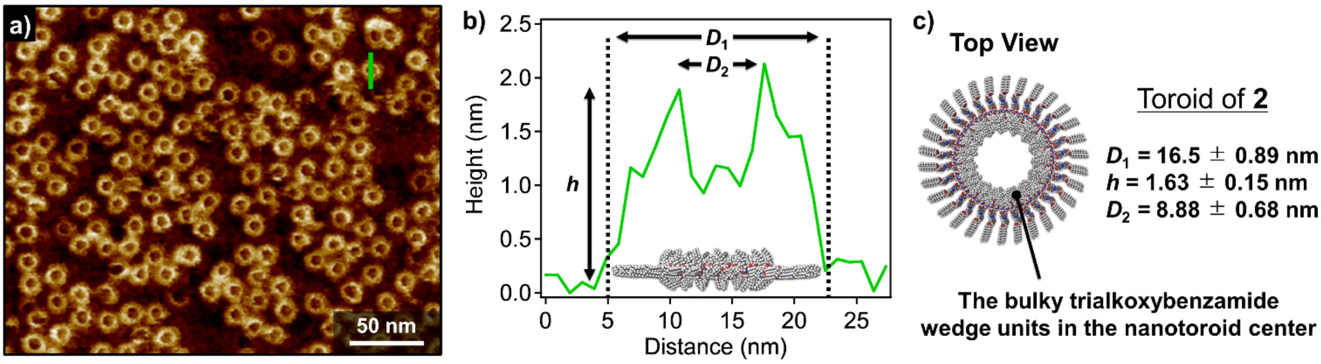

Figure S9. a) Magnified AFM image of nanotoroids of **2**. b) AFM cross-sectional analysis of a nanotoroid of **2** along the green line in (a). c) Schematic representation of the proposed toroidal nanostructures of **2**. Bulky wedge groups located inside the nanotoroid of **2**.

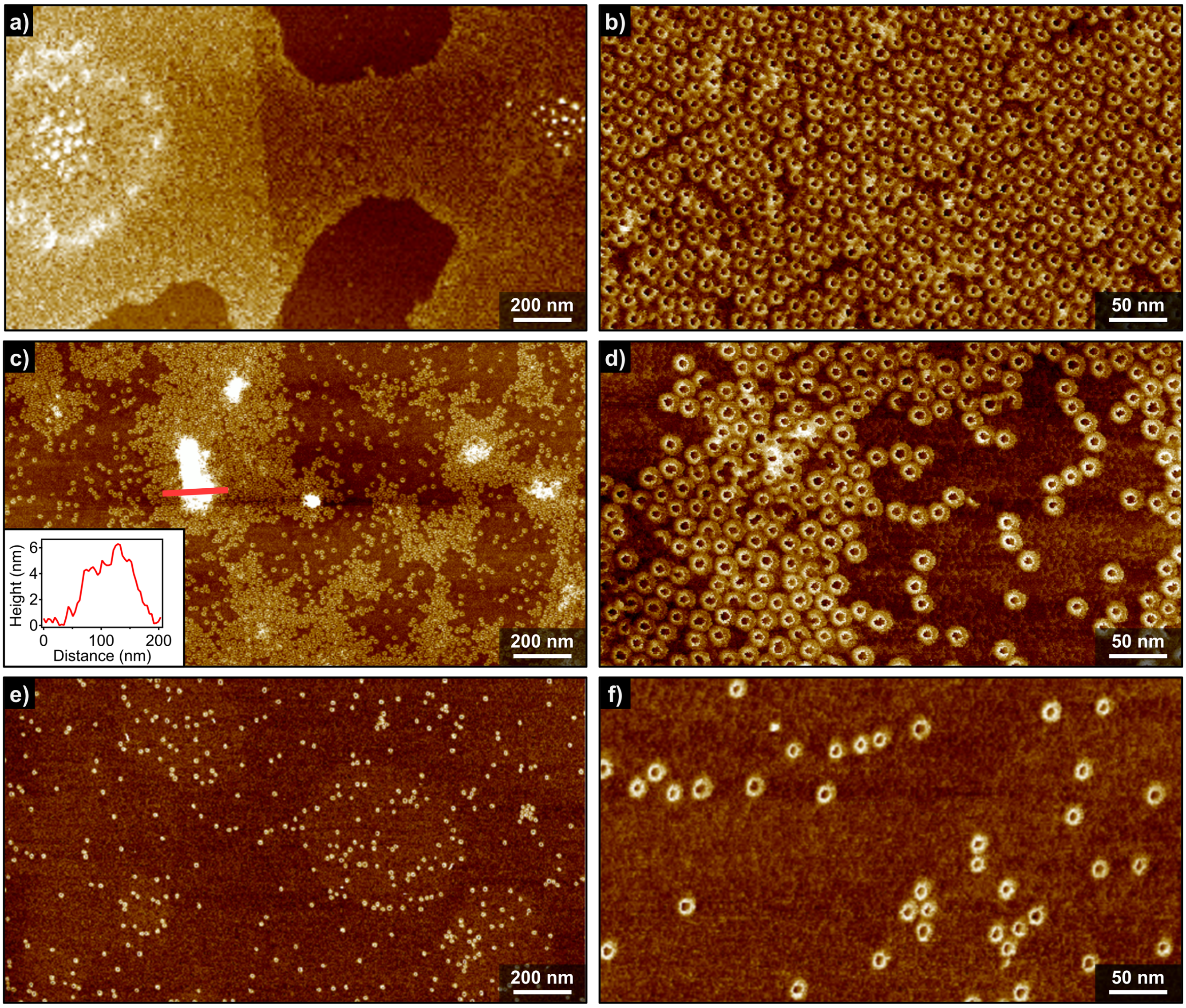

Figure S10. a–f) AFM images of nanotoroids of **(*S*)-2** in (a,b) MCH, (c,d) *n*-octane and (e,f) *n*-dodecane (c_T = 100 μM) obtained by cooling the solution to 10 ºC. Inset in (c) shows a cross-sectional analysis along the red line in (c).

Supporting Discussion: As described in the main text, AFM images of a–d) suggests the same degree of nanotoroid is formed in *n*-octane as in MCH. The difference is that the nanotoroids formed in *n*-octane were more aggregated to form "islands" with heights higher than those of nanotoroids by 4–6 nm in places. This can be rationalized by the volatilities of solvents and the heat of vaporization, defined as the amount of heat required to vaporize a definite quantity. It is often referred to as the latent heat of vaporization, ΔH_v, which is 35.359 kJ mol^{-1} for MCH, 41.49 kJ mol^{-1} for *n*-octane, and 61.287 kJ mol^{-1} for *n*-dodecane, respectively.[S4] Accordingly, the most volatile MCH allows a kinetic homogeneous adsorption of nanotoroids to HOPG. According to this line, the least volatile *n*-dodecane should result in the heaviest aggregation of nanotoroids as a thermodynamic process. However, our thorough investigation of the spin-coated films prepared from *n*-dodecane solution confirmed an evidently low yield of nanotoroids in this solvent. From this finding, we have proposed the contribution of the intramolecularly hydrogen-bonded species in *n*-dodecane as discussed in the main text.

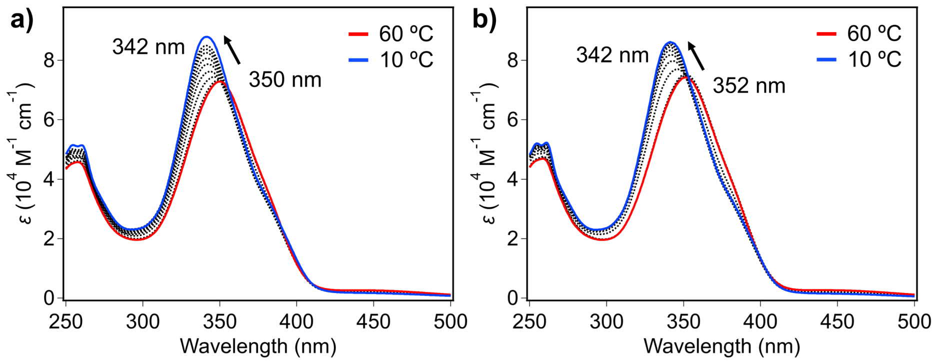

Figure S11. a,b) VT-UV-Vis spectra of **2** in (a) *n*-octane and (b) *n*-dodecane (c_T = 300 μM) upon cooling from 60 to 10 ºC at a rate of 1 ºC min^{-1}. The temperature interval between spectra was 5 ºC.

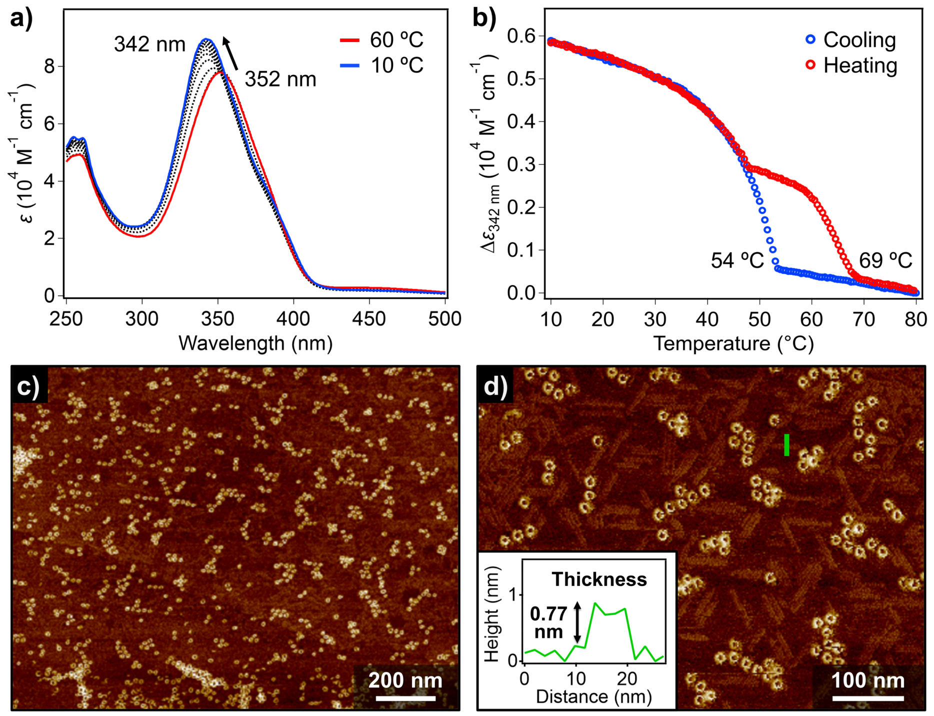

Figure S12. a) VT-UV-Vis spectra of **2** in *n*-dodecane (c_T = 300 μM) at different temperatures. The temperature interval between spectra was 5 °C. b) Plot of the variation of the molar extinction coefficient (ε) of **2** at 342 nm versus temperature extracted from VT-UV-Vis spectra. c,d) AFM images of nanotoroids formed by **2** in dodecane (c_T = 100 μM). In d), molecular-level thin linear assemblies are clearly observed. These assemblies have thickness around 0.77 nm (inset).

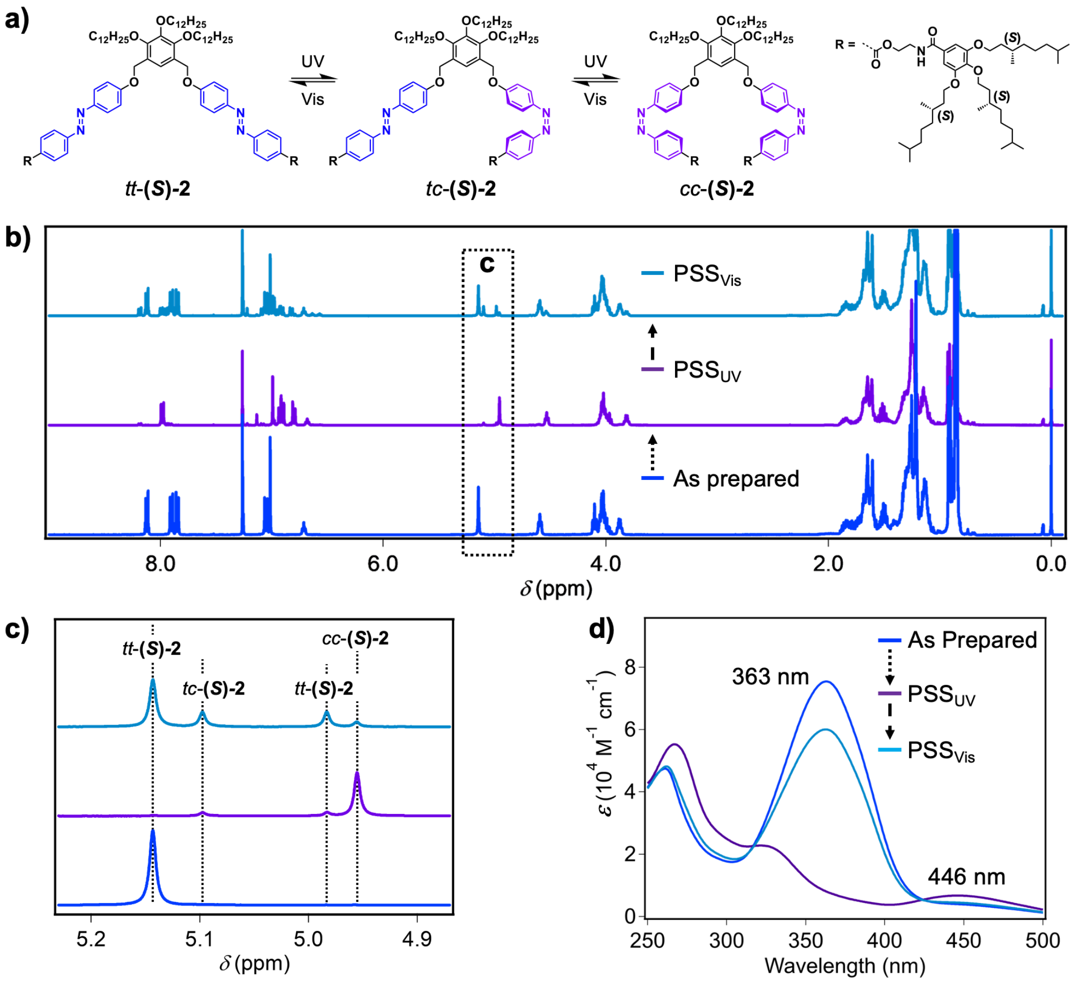

Figure S13. a) Chemical structures of *tt*-**(*S*)-2**, *tc*-**(*S*)-2** and *cc*-**(*S*)-2**, respectively. b) ^{1}H NMR spectra of **(*S*)-2** in $CDCl_3$ (c_T = 5 mM) at 20 °C before (blue spectrum), after irradiation with UV light for 30 min (PSS_{UV}, purple spectrum) and with visible light for 15 min (PSS_{Vus}, sky-blue spectrum). c) Partial ^{1}H NMR spectra of the spectra in (b), wherein the integration of the benzylic proton signals of *tt*-**(*S*)-2** *tc*-**(*S*)-2**, and *cc*-**(*S*)-2** shows *tt*-**(*S*)-2**:*tc*-**(*S*)-2**:*cc*-**(*S*)-2** = 1:5:94 for PSS_{UV}, = 71:23:6 for PSS_{Vis}, revealing 4:96 and 82:18 *trans*:*cis* ratio of azobenzene moieties, respectively. d) UV-Vis absorption spectra of **(*S*)-2** in $CHCl_3$ (c_T = 300 μM) at 20 °C recorded as-prepared (blue spectrum), after irradiation with UV light for 30 s (PSS_{UV}, purple spectrum), and with visible light for 1 min (PSS_{Vis}, sky-blue spectrum), respectively. The absorption intensity at 363 nm shows an 89% decrease upon irradiation with UV light, which corresponds to the 96% *trans*→*cis* isomerization according to the ^{1}H NMR analysis in (b). This relationship has been used as a reference to calculate photoisomerization yield of azobenzene moieties in nanotoroids in MCH.

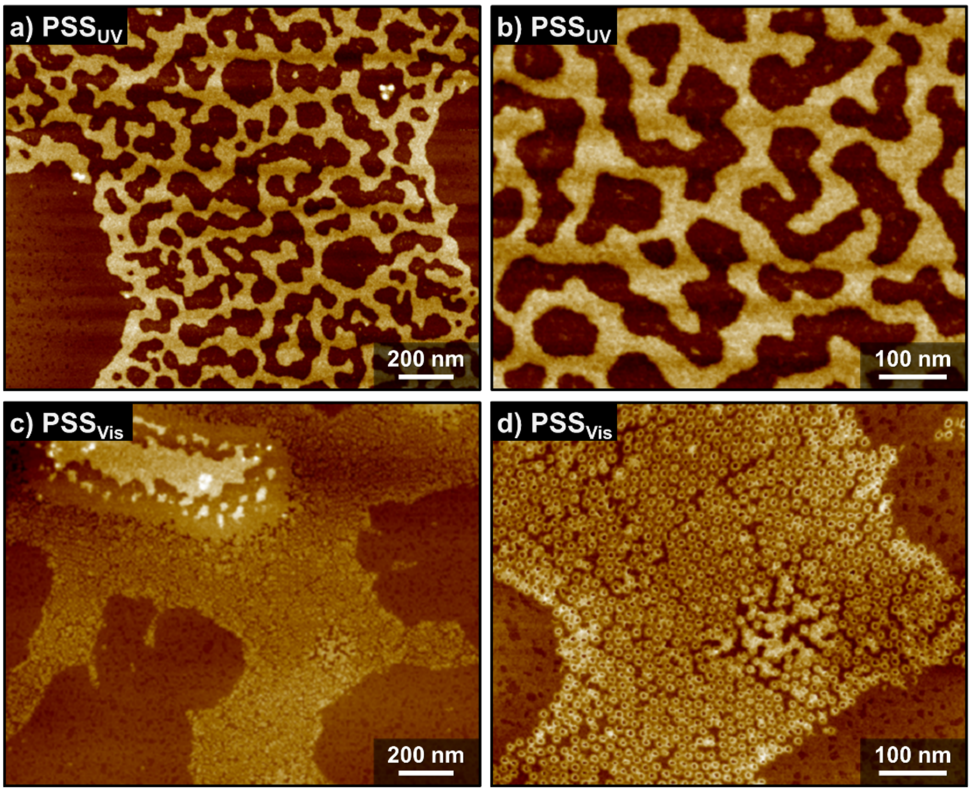

Figure S14. a–d) AFM images of nanostructures of **(*S*)-2** in MCH (c_T = 100 μM) obtained by irradiation with (a,b) UV light to the as-prepared MCH solution and (c,d) with visible light to the PSS_{UV} solution.

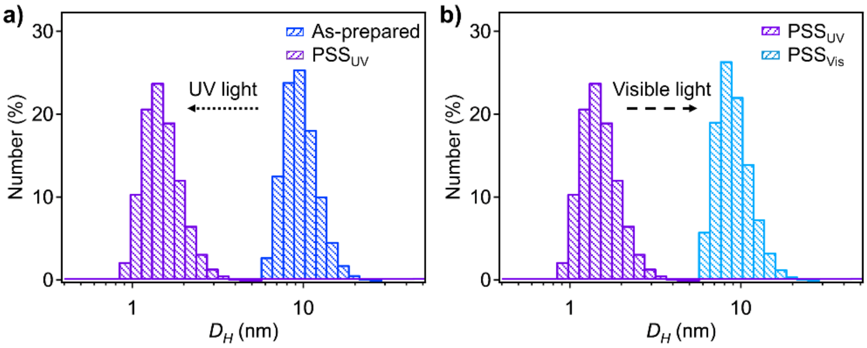

Figure S15. a,b) DLS size distribution of **(*S*)-2** in MCH (c_T = 100 μM) at 20 °C (a) before UV-light irradiation (blue bars) and at PSS_{UV} (purple bars), and (b) at PSS_{UV} (purple bars) and at PSS_{Vis} (sky-blue bars), respectively.

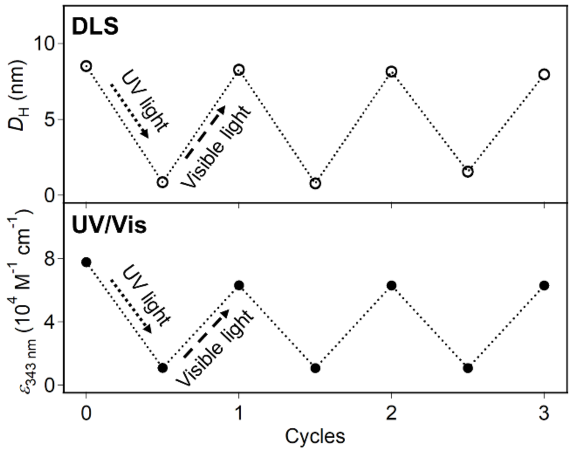

Figure S16. Change of the average D_H (upper) and ε at 342 nm (lower) of **(*S*)-2** in MCH (c_T = 300 μM) at 20 ºC upon UV- and visible-light irradiation cycle.

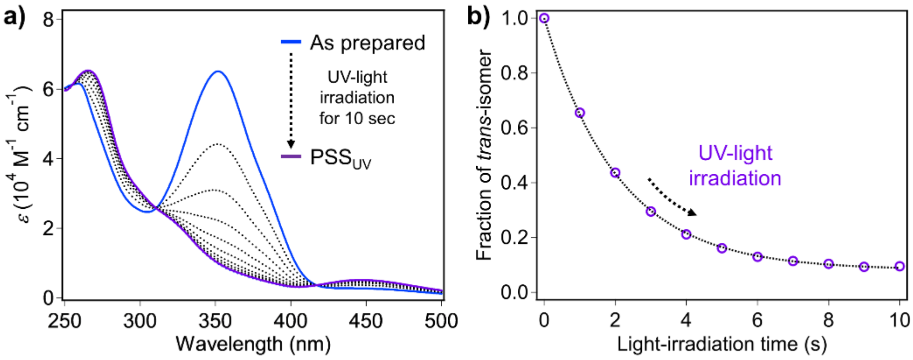

Figure S17. a) UV-Vis spectral change of monomeric **(*S*)-2** in MCH (c_T = 40 μM) at 20 °C upon UV-light irradiation for 10 s (from blue to purple spectra) to reach PSS_{UV}. The time interval between spectra was 1 s. The absorption intensity at 351 nm shows an 84% decrease upon irradiation with UV light, of which value is identical to that of the UV-irradiation experiment to a nanotoroid sample (c_T = 300 μM). b) Plot of the change in the mole fraction of *trans*-isomer as a function of UV-light irradiation time. The black dotted line was the best fitting curve by a simple mono-exponential equation[S5].

4.- References

[S1] T. Saito, S. Yagai, *Org. Biomol. Chem.* **2020**, *18*, 3996–3999.

[S2] F. Aparicio, L. Sánchez, *Chem. Eur. J.* **2013**, *19*, 10482–10486.

[S3] M. M. J. Smulders, A. P. H. J. Schenning, E. W. Meijer, *J. Am. Chem. Soc*. **2008**, *130*, 606–611.

[S4] J. A. Riddick, W. B. Bunger, T. K. Sakano, *Organic Solvents: Physical Properties and Methods of Purification*: John Wiley and Sons: New York, 1986.

[S5] T. Saito, S. Yagai, *Eur. J. Org. Chem.* **2020**, 2475–2478.

Author Contributions

Jorge S. Valera (equal); Hironari Arima (equal), Cristina Naranjo, Takuho Saito, Natsuki Suda: investigation, methodology, visualization, writing. Rafael Gómez: investigation, writing; Shiki Yagai, Luis Sánchez: conceptualization, writing, supervision, funding acquisition.
